# Will the COVID-19 pandemic slow down in the Northern hemisphere by the onset of summer? An epidemiological hypothesis

**DOI:** 10.1007/s15010-020-01460-1

**Published:** 2020-06-23

**Authors:** Alexander Dzien, Christine Dzien-Bischinger, Monika Lechleitner, Hannes Winner, Günter Weiss

**Affiliations:** 1 Practice for General Internal Medicine, Innsbruck, Austria; 2 Department of Internal Medicine, District Hospital Hochzirl-Natters, Innsbruck, Austria; 3 Department of Social Sciences and Economics, University of Salzburg, Salzburg, Austria; 4 Department of Internal Medicine II, Medical University of Innsbruck, Anichstr. 35, 6020 Innsbruck, Austria

**Keywords:** COVID-19, Epidemiology, Respiratory viral infection, Influenza, Seasonality, Pandemic

## Abstract

The COVID-19 pandemic has affected most countries of the world. As corona viruses are highly prevalent in the cold season, the question remains whether or not the pandemic will improve with increasing temperatures in the Northern hemisphere. We use data from a primary care registry of almost 15,000 patients over 20 years to retrieve information on viral respiratory infection outbreaks. Our analysis suggests that the severity of the pandemic will be softened by the seasonal change to summer.

The infection caused by the human corona virus COVID-19 (SARS-CoV2) resulted in a worldwide pandemic affecting several million people and causing severe disease and fatality mostly based on virus mediated lung failure [1, 2]. Thus far, no effective therapy is available and, therefore, contact precautions, hygiene measures, contact tracing, social distancing and quarantine are the methods of choice to control the spread of this infection [3, 4]. However, epidemics with respiratory virus such as not only influenza but also human corona viruses are prevalent in the Northern hemisphere over several months during the cold season and then disappear whereas influenza remains prevalent in tropical regions throughout the whole year [5–7]. The reasons for this decline are still incompletely understood. We thus questioned whether or not the COVID-19 pandemic will be in part slowed down by the change from the cold to the warm seasons in the Northern hemisphere.

To assess local epidemics from respiratory infections over the course of a season, we retrospectively analyzed the records of 15,336 patients (8907 women and 6429 men) who visited a primary care internal medicine practice in Innsbruck, Austria, between July 1, 1999, and March 15, 2020. Overall, our dataset included 145,740 diagnoses, of which 16,317 are assigned to acute respiratory diseases according to the International Classification of Diseases (ICD-10; www.icd.who.int/browse10/2019/en). In particular, we included viral and bacterial infections classified into diagnostic groups A and B as well as infectious diseases of the respiratory tract of groups J (pulmonology), H (ear, nose and throat) and R (general symptoms consistent with infections such as dyspnea or fatigue). Based on this data, we first counted the number of patients who sought medical help because of serious respiratory infections. Second, we related these counts to the total number of diagnoses within each month to obtain the share of viral infections. In the denominator of this ratio, we excluded malignancies (group C), neoplasia (D), diabetes mellitus 2 (E-11.9), arterial hypertension (I-10) and ischemic heart diseases (I-20). As the mentioned practice was sometimes closed due to holiday breaks, the share of viral infections might provide a more suitable figure on seasonal patterns than the simple count measure.

Figure 1 plots the counts of respiratory diseases over the course of the years. It shows a strong seasonal pattern with peaks around the winter months December to March. The strongest upward deviations are observed in the years between 2008 and 2010 and for 2015. Figure 2 depicts the share of respiratory diseases and pools the information from Fig. 1 into one graph. The smoothed lines represent a non-linear time trend based on year-wise regressions with the relative contributions of respiratory diseases per month as the dependent variable. In particular, we included a linear and a quadratic time trend, which allows to calculate predicted values and, in turn, the hump-shaped graphs in Fig. 2. We also used more flexible functional forms, e.g., higher order polynomials, but it turned out that the quadratic specifications performed well in terms of overall fit; a pooled regression over all seasons led to an *R*^2^ of about 60%. All the calculations are carried out with Stata (version 16).

**Fig. 1:**
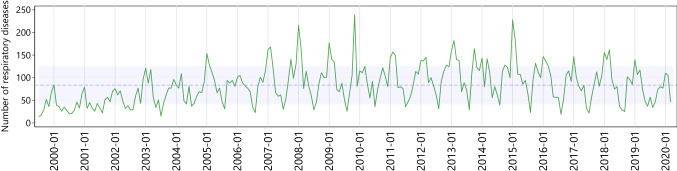
The total number of infectious respiratory diseases (green line) over a time period of 20 years (1999–2020). The dashed line indicates the overall monthly mean of diagnoses over this time period (84.5 ± 41.6), and the shaded area shows the upper and lower bounds of one standard deviation around this mean

**Fig. 2:**
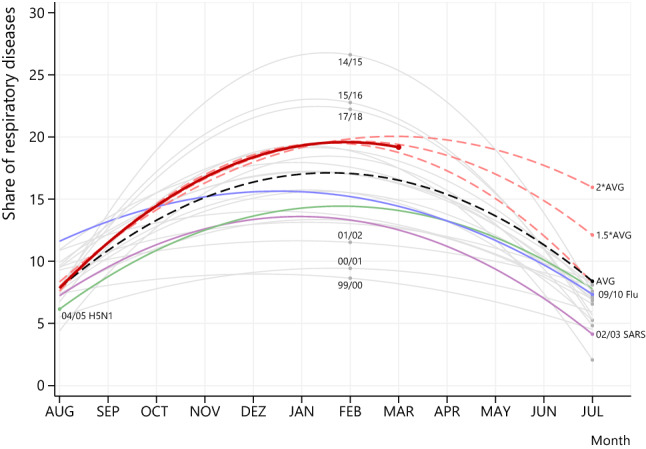
The relative number of infectious respiratory diseases over a time period of 20 years (1999–2020). For each month, the total number of respiratory diseases is related to the total number of diagnoses in the physician practice (*y*-axis). The figure plots the seasons in which previous respiratory infections have been recorded (02/03 SARS-CoV, purple line; 04/05 H5N1, green line; 09/10 pandemic flu H1N1v, blue line), and those where this was not the case (grey lines). Regarding the pattern of respiratory diagnoses in the season 2019/20, we first used the monthly data until March 15 (red line). A red line is marked here and is a solid line from Aug to Mar. We further fitted a “potential” seasonal development of respiratory diseases for three scenarios (dashed red lines): (i) one where the share of respiratory diseases in July 2020 is equal to the average of respiratory diseases (indicated by “AVG”) over the month of July of the whole sample period of 20 years; (ii) one where this average is 1.5 times higher; (iii) one where this average is two times higher

The colored lines plot the seasons in which previous pandemic respiratory infections have been recorded (02/03 SARS-CoV, purple line; 04/05 H5N1, green line; 09/10 pandemic flu, blue line) [8] but not showing the absolute contributions of these pandemic infections to the total number of respiratory infections. The grey lines indicate seasons where no specific pandemic respiratory infections are recorded. In line with Fig. 1, we can see that the peak of respiratory diseases (including putatively COVID-19) lies between February and April 2020 followed by a prominent and sustained reduction towards summer.

If COVID-19 would behave similar to other respiratory viruses causing respiratory infections including human corona viruses which peak during winter time and early spring, there is hope that the COVID-19 pandemic can be slowed down by this seasonal trend [7, 9]. In those seasonal infections, no herd immunity is achieved during a specific season [7]. This would go in a line with the duration of the SARS-CoV-1 epidemic in 2002/2003, which also started in China, peaked in February to April and was terminated in summer 2003 although strict contact precaution measures were likewise the main secret of success [9]. However, pandemics with new viruses such as the influenza H1N1v can circulate independent of typical respiratory viral seasons throughout the whole year [10]. In line with this, the pandemic “Spanish flu” presented even with a summer peak in Scandinavia in the year 1918 preceding the major outbreak in the cold season of 1918/1919 in this area [11]. This might be the only one published exception of an influenza summer epidemic. The next few months will provide a definitive answer to which scenario will hold true for the control of COVID-19 infections and how changes of temperature and social behavior will impact on the control of this pandemic.
